# Monolithic Polyepoxide Membranes for Nanofiltration Applications and Sustainable Membrane Manufacture

**DOI:** 10.3390/polym16182569

**Published:** 2024-09-11

**Authors:** Mackenzie Babetta Anderson, Riley A. Danna, Clayton French, Jishan Wu, Markus N. Thiel, Zhiyin Yang, Eric M. V. Hoek, Richard B. Kaner

**Affiliations:** 1 Department of Chemistry and Biochemistry, University of California, Los Angeles (UCLA), Los Angeles, CA 90095, USA; 2 Department of Civil & Environmental Engineering, University of California, Los Angeles (UCLA), Los Angeles, CA 90095, USA; 3 Energy Storage & Distributed Resources Division, Lawrence Berkeley National Laboratory, Berkeley, CA 94720, USA; 4 Department of Materials Science and Engineering, University of California, Los Angeles (UCLA), Los Angeles, CA 90095, USA

**Keywords:** epoxy resin, carbon fiber-reinforced composite, membrane, nanofiltration, carbon fiber recycling, polymer degradation, interfacial polymerization

## Abstract

The present work details the development of carbon fiber-reinforced epoxy membranes with excellent rejection of small-molecule dyes. It is a proof-of-concept for a more sustainable membrane design incorporating carbon fibers, and their recycling and reuse. 4,4′-methylenebis(cyclohexylamine) (MBCHA) polymerized with either bisphenol-A-diglycidyl ether (BADGE) or tetraphenolethane tetraglycidylether (EPON Resin 1031) in polyethylene glycol (PEG) were used to make monolithic membranes reinforced by nonwoven carbon fibers. Membrane pore sizes were tuned by adjusting the molecular weight of the PEG used in the initial polymerization. Membranes made of BADGE-MBCHA showed rejection of Rose Bengal approaching 100%, while tuning the pore sizes substantially increased the rejection of Methylene Blue from ~65% to nearly 100%. The membrane with the best permselectivity was made of EPON-MBCHA polymerized in PEG 300. It has an average DI flux of 4.48 LMH/bar and an average rejection of 99.6% and 99.8% for Rose Bengal and Methylene Blue dyes, respectively. Degradation in 1.1 M sodium hypochlorite enabled the retrieval of the carbon fiber from the epoxy matrix, suggesting that the monolithic membranes could be recycled to retrieve high-value products rather than downcycled for incineration or used as a lower selectivity membrane. The mechanism for epoxy degradation is hypothesized to be part chemical and part physical due to intense swelling stress leading to erosion that leaves behind undamaged carbon fibers. The retrieved fibers were successfully used to make another membrane exhibiting similar performance to those made with pristine fibers.

## 1. Introduction

This work demonstrates the use of a carbon fiber (CF)-reinforced epoxy composite in membrane applications, including subsequent extraction and reuse of the carbon fibers. It blends the areas of polymer chemistry, membrane science, composite design, and carbon fiber recycling. The membranes are designed to use readily available chemical feedstocks and have planned end-of-life while simultaneously addressing the need to create chemically tolerant membrane materials that can simplify industrial membrane processes. By using only a simple nonwoven veil that can be made from waste materials from CF reinforced epoxy manufacturing across industries, along with a two-part epoxy resin cast from polyethylene glycol (PEG, a green solvent) to fabricate a complete membrane, the achievement is a highly tunable membrane system that avoids solvent intensive interfacial polymerization (IP) and reduces the total number of membrane components. The overall complexity of a membrane is reduced while water flux and rejection of small-molecule dyes are competitive with commercial membranes and comparable lab-scale prototypes. In this section, factors that drive interest in creating new types of membrane materials are introduced, followed by past work on epoxy-based membranes, their use in different applications, and their shortcomings. In particular, the need to reduce solvent use and the environmental impacts of membrane modules are presented. The importance of CF composites and their recycling is introduced, along with how CF may be an important approach to sustainable membrane manufacturing. Lastly, performance data for the most relevant commercial and lab-scale membranes are provided, along with the limitations of such comparisons.

**Figure 1:**
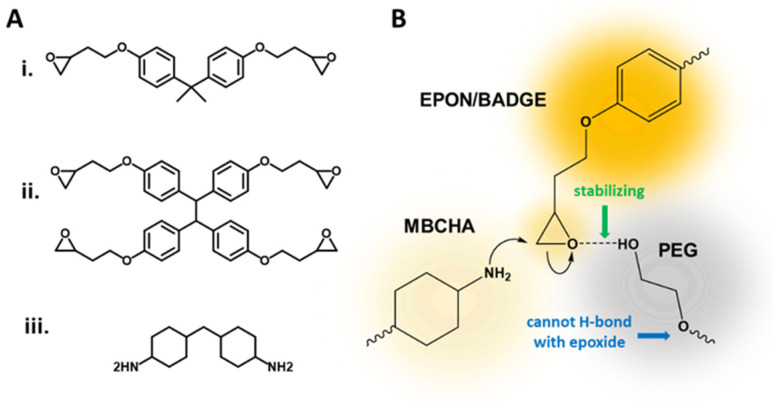
(**A**) Three structural monomers used in this work: (**i**) BADGE, (**ii**) EPON, and (**iii**) MBCHA; (**B**) Stabilization of the epoxide ring opening via hydrogen bonding with the terminal -OH of a polyethylene glycol.

Polyamide (PA) water purification membranes made by solvent-intensive interfacial polymerization of meta-phenylene diamine (MPD) and trimesoyl chloride (TMC) are the industry standard and the most thoroughly studied and optimized membrane materials, but cannot withstand harsh chemical environments, including common disinfectants [1,2,3]. Therefore, energy-intensive pretreatment of feed solutions is required before contact with these membranes. To address this shortcoming, Verbeke et al. proposed the novel synthesis of a polyether epoxide thin film composite (TFC) membrane with an EPON™ Resin 1031 (EPON) active layer (Figure 1A(ii)), translating epoxies that are traditionally made via bulk syntheses to an interfacial polymerization [4]. Lacking readily cleavable amide bonds, they demonstrated how an EPON active layer was highly resistant to caustic environments, while, although the poly(epoxyether) layer was undamaged, the polyimide (PI) support membrane they used was susceptible to degradation, and thus they proposed that an alternative support membrane be used. In a later publication, Verbeke et al. demonstrated that key to the selectivity of these TFC membranes is the “solvent annealing” of the PI support layer and that the poly(epoxyether) active layer only plays a role for the separation of very small organic molecules, leaving much room for the optimization of this material system for use in membranes [5]. They later replaced the PI with polyacrylonitrile and crosslinked the epoxy selective layer to achieve greater stability and rejection [6]. In past work, we similarly addressed the stability of membranes in caustic and chlorinated environments using epoxy-supported polybenizimidazoles (PBIs) [7]. While the use of an epoxy support was novel, when it came to rejection of solutes, the focus was on the chemistry of the PBI active layer. To advance the application of poly(epoxyethers) to separations, here we present a novel “all-epoxy” membrane, addressing both the need for a chemically robust support membrane and other environmental factors such as recyclability.

Structures of monomers used in this work are given in Figure 1A. Poly(epoxyether) composite membranes made from bisphenol-A-diglycidyl ether (BADGE, Figure 1A(i)) and methylenebis(cyclohexylamine) (MBCHA, Figure 1A(iii)) in the presence of polyethylene glycol can make effective support layers for asymmetric membranes [7]. A polybenzimidazole-polystyrene sulfonic acid-based active layer upon a finely tuned poly(epoxyether) support can be used to separate NaCl from water under brackish water concentrations [7]. This epoxy chemistry was initially tuned to be used for a variety of filtration applications by adhering to thin films of various active layers, using a method called Thin-Film Lift Off (T-FLO) [7]. The porosity of the poly(epoxyether) membranes, and as a result the compaction, water flux through, and retention of a membrane, is controlled via the molecular weight (*M_w_*) of the polyethylene glycol (PEG) solvent, indirectly controlling the number of hydroxyl end-groups that have a stabilizing/catalytic effect during the ring-opening of the epoxide, as illustrated in Figure 1B [7]. In subsequent publications, we demonstrated that the T-FLO technique is amenable to create TFC membranes with graphene oxide (GO) and polybenzimidazole (PBI) active layers for nanofiltration membranes with both organic and aqueous feeds [7,8,9]. Still, the role of rejection by just the epoxy was not thoroughly considered. Here we demonstrate that the thin-film active-layer may not even be necessary to achieve a comparably low molecular weight cut off (MWCO), below 400 Da, for small-molecule dyes in aqueous feeds. Using monolithic EPON and BADGE-based epoxy resins, apart from T-FLO or interfacial polymerization, membranes can achieve MWCO below 400 Da without either an active layer or a woven support. Still, how does this advance the sustainability or improve upon the operation of a chemical treatment process?

For commercial applications, hundreds of square feet of flat sheet membranes are wound into membrane elements that are placed into pressure vessels. These are complex materials consisting or membrane active and support layers, the plastic feed/permeate channel spacers that separate membrane sheets and allow permeation, the collection tube, and the fiber glass case [10]. Large membrane-based water treatment plants can contain up to tens of thousands of membrane elements per facility [11,12]. A typical 8-inch spiral wound membrane module has ~400 square feet of active layer surface area [11,13,14,15,16,17]. Currently, once these membranes stop performing, they are disposed of in landfills due to the difficulty of recycling. Lawler et al. explain how membranes could be diverted from landfills through reusing membranes for less intensive separations; extensive processing to separate recyclable components; and finally, incineration and gasification of what is left for electricity generation [10]. They conclude that these methods may offset some of the environmental impacts of manufacture and transportation. Still, factors such as the use of petroleum-based solvents for membrane fabrication, etc., make it difficult to gauge the total environmental impact of waste produced during membrane plant operation [10]. Therefore, due to the expense of disassembly of the membrane modules to obtain their respective parts for recycling/reuse, membranes with fewer, more reusable components, such as those presented in this work, are attractive.

The interest in using carbon fibers in the development of new types of membranes are their relative inertness reducing their chemical interference with the polymer systems of interest, as well as their lack of thermal expansion upon thermal curing or exposure to solvents. In terms of sustainable manufacturing, CF is not inherently the “greenest” option. Making carbon fibers is an energy intensive process. But, nonwovens like those employed in this work can be made from scrap carbon fibers from other industries, which can be up to 40% of the total mass of waste generated in CF composite manufacture [18,19]. Therefore, the use of carbon fiber nonwovens for membranes is upcycling of an industrial byproduct.

Lawler et al. also attempt to quantify the relative environmental impact of each component of the membrane, including the module pressure vessel, the support membrane and active layer, and spacers; the most consequential component being the polyethersulfone support and the PA active layer [10]. The membranes in this work consist of a smooth-side and a side characterized by the morphology of the carbon fiber veils used as the support matrix. Because the fiber-facing side is inherently sturdy and forms random channels, it could create its own channels for the collection of permeate. This may reduce the need for a feed-spacer in a spiral wound module, which may even further incentivize the development of more novel membrane processes.

If the need for interfacial polymerization (IP) or nonsolvent immersion precipitation (NIPS) is eliminated for rejection of solutes in the MWCO range of interest, this work could address two major challenges facing the membrane industry: the large volumes of organic solvents used in synthesis/fabrication and the low potential for recyclability of membranes as they are currently manufactured. Membrane fabrication, whether formed by NIPS or IP, requires the use of large amounts of organic solvents for dissolving polymer solutions and dissolving monomers, respectively. This use has negative effects on health and equipment, while reducing the sustainability of the field [20]. Attempts to mitigate this problem include minimizing solvent use [21], employing safer solvents, using fewer volatile solvents [22,23], and eliminating the need for solvents altogether [22,23]. The epoxy system developed in our previous work requires only PEG as a solvent and porogen and a water bath for PEG removal. PEG is generally considered safe, appearing in many products including cosmetics [24], laxatives [25], and contact lenses [26]. Additionally, PEG can be removed from wastewater using nanofiltration membranes, unlike other solvents that require distillation to separate them out from solvent mixtures [27]. Therefore, monolithic membranes using only PEG and water could be a solution for reducing the environmental impact of manufacturing ultrafiltration and nanofiltration membranes.

While epoxy membranes may be well suited to withstand harsh conditions, and thus reduce the need for extensive pretreatment, one challenge posed by all heavily crosslinked materials is their inability to be recycled. Carbon fiber-reinforced epoxies are used in many products because of their combined high strength and light weight [18,19,28,29,30]. But because carbon fiber is expensive, due largely to the petroleum-based precursors, its applications are often limited to high-value products [29]. This has led to an abundance of research in the area of making carbon fiber from more sustainable precursors [29,30]. Still, used carbon fiber composites typically end up in landfills due to the difficulty of breaking down the composite matrices such that recycling of fibers (e.g., by mechanical means) seriously degrades the fibers [28,29]. The development of techniques to improve the recyclability of carbon fiber-reinforced composites, while maintaining the integrity of the virgin materials, has become increasingly important with the growing use of carbon fibers and the environmental imperative to reduce waste sent to landfills and industry’s overall carbon footprint [28,29,30,31]. Processes that require relatively low energy input (below that of making new fibers from virgin materials) and those that do not employ toxic solvents are most desirable. The membranes in this work can be separated into a solid polymer byproduct and high-quality carbon fibers by treatment with commercial bleach. The fibers can then be reused to make a new high-rejecting membrane. Beyond the scope of this work, it has been shown that similarly degraded solid polymer byproducts may be combined with epoxy resin to form a similarly strong resin upon optimization [32,33]. Therefore, the polymer byproduct may feasibly be useful in the remaking of membranes, along with the carbon fibers. Lastly, work by Kizaki et al. has demonstrated a continuous manufacturing technique. By using a similar pre-impregnated nonwoven, it is likely a similar technique could be used to create a roll-to-roll process for the membranes proposed in this work [34].

As the title implies, the monolithic nature of the membranes here makes them novel, but the importance and prevalence of interfacial polymerization is not neglected herein. The membranes in this work can also be amenable to these processes, but, with these epoxy systems (BADGE/EPON) like past findings, the role of the support in rejection is higher than that of the active layer for the MW range of interest for nanofiltration [5,7]. The primary factors in formation of an active layer atop a support include the support layer’s pore sizes, surface porosity (i.e., number of pores per surface area), pore size distribution, and the relative hydrophobicity/hydrophilicity [35,36,37,38,39]. For a traditional PA membrane, these factors affect the diffusion of reactants (MPD and TMC) and products (HCl) to and from the organic–aqueous interface, affecting both the kinetics of film formation and the resulting physical and chemical properties. Here, we show how membranes made from BADGE-MBCHA can be used to support an EPON polyether epoxide thin film through interfacial polymerization. However, without further optimization, the membranes did not perform much better than those made directly from EPON-MBCHA that lacked an interfacially polymerized thin film.

All-epoxy membranes and other epoxy-supported membranes in the literature have very different rejection profiles than state-of-the-art PA membranes. Flux through Synder Filtration’s commercial NFG PA/TFC membranes with MWCO of 600–800 Da is about 13.6 LMH/bar and NFW PA/TFC with MWCO 300–500 Da is about 11.4 LMH/bar [13,14]. While these membranes are reported to reject 50% MgSO_4_ and 97% of MgSO_4_, respectively (much higher than this work), they have rejection of Lactose (MW = 342.3 g/mol) of 98.5% and 60%, respectively. A more comparable membrane made from an epoxy support with an IP epoxy active layer had permeance of 20/80 DMF/water of 0.04–0.5 LMH/bar with 99.2% rejection of 17.5 μM Rose Bengal (MW = 973.67 g/mol) [15]. An earlier report of an EPON active layer atop a polyimide support was reported to have a flux of 1.5 LMH/bar and 89.5% rejection of Rose Bengal [4]. Therefore, the flux and rejection in this work is on par with commercial membrane rejection of *organic* compounds as well as permeability, but not salt rejection. Still, when it comes to rejection of dyes, because lab scale testing is done in a dead-end format, a direct comparison to the literature is difficult due to lack of reporting of recovery (amount of permeate recovered compared to initial volume of feed) in the literature. Although measures are often taken (such as stirring) to prevent the effect of concentration polarization, its effect will inevitably have an impact on the reported rejection values. If less permeate is collected, the rejection will be lower.

Portions of this article relevant to the synthesis, characterization, and dye rejection of membranes appear in Chapter 2 of the lead author’s Ph.D. thesis [40].

## 2. Materials and Methods

### 2.1. Materials

4′4 methylene bis cyclohexylamine (MBCHA) was obtained from Acros Organics (ThermoFisher Chemical, Loughborough, UK) and used without further preparation. Bisphenol-A-diglycidyl ether (BADGE), polyethylene glycol (PEG) (MW 200, MW 300, and MW 400), and tetramethyl N,N,N’,N’ tetramethyl-1,6 hexane diamine (TMHD), Methylene Blue, and Rose Bengal were purchased from Sigma-Aldrich (St. Louis, MO, USA) and used as packaged. Tetraphenolethane tetraglycidylether, EPON™ Resin 1031 (EPON), pellets were obtained from Hexion (Hexion Inc., Columbus, OH, USA) and used without further treatment. Epoxyease mold-release spray was obtained from Slide Products Inc. (Wheeling, IL, USA) Nonwoven carbon fiber veil derived from polyacrylonitrile (PAN) was obtained from Fibre Glast (Brookville, OH, USA) (product number 1064) and used without modification. Sodium hypochlorite (8.25%, 1.11 M) was used in the form of concentrated Clorox Commercial Bleach (The Clorox Company, Oakland, CA, USA).

### 2.2. Membrane Synthesis

A series of membranes were made from BADGE and MBCHA, as described in detail in reference [7] (McVerry et al.). Water flux through the membranes was measured gravimetrically and rejection of Rose Bengal and Methylene Blue dyes were determined spectrophotometrically. After evaluating the standalone epoxy membranes, an interfacially polymerized EPON active layer was added onto each membrane. When these membranes were viewed under via SEM, the successful formation of the active layer was observed. Tests for flux and rejection were repeated for these membranes. Finally, the BADGE monomers were replaced with EPON to yield more crosslinked structures (see Figure S1 for the structures). These membranes were similarly tested for flux and rejection.

### 2.3. Epoxy Formation

The formulations for all membranes used in this study are summarized in Table S1.

For making BADGE (diglycidyl ether) based membranes: In a 20 mL glass scintillation vial, 330 mg of MBCHA was dissolved in 3.25 g of PEG 200, 300, or 400. To the solution was then added 1.0 g of BADGE. The mixture was stirred at 750 rpm for 3 h.

For making EPON (tetraglycidylether) based membranes: In a 20 mL glass scintillation vial, 1.0 g of EPON was dissolved in 3.25 g of PEG 200, 300, 400, or a combination thereof. The solution was gently heated and stirred overnight to achieve complete dissolution. At room temperature, 330 mg of MBCHA was added to the solution and left stirring at 750 rpm for 3 h. Over the course of stirring, the solution becomes more viscous.

### 2.4. Membrane Fabrication

**Figure 2:**
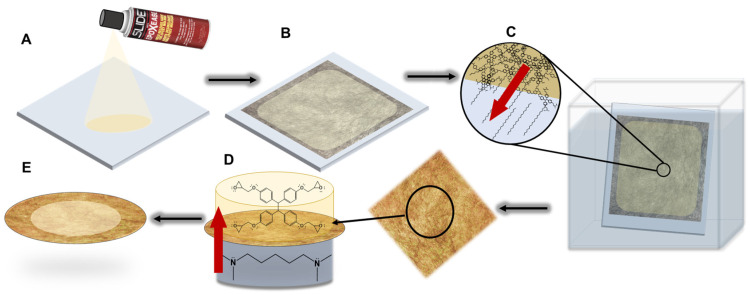
(**A**) A glass substrate is coated with Epoxease mold release. (**B**) A carbon fiber veil is placed onto the support and the epoxy resin formulation is poured onto the veil followed by curing at 130 °C for 4 h. (**C**) Cooled, cured membranes are then placed into a water bath and PEG leaches from the polymer. (**D**) A membrane punchout is next placed into a hand-made interfacial polymerization reactor with aqueous TMHD in the bottom chamber and EPON in toluene in the top chamber. Red arrow indicates direction of initiator diffusion from aqueous to organic layer. (**E**) The membrane with an EPON active layer is complete.

**Figure 3:**
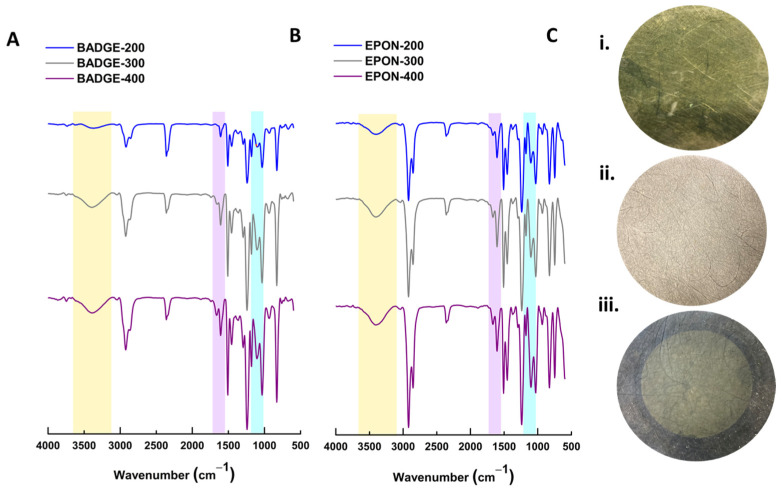
(**A**) ATR-IR spectra for membranes made from BADGE and MBCHA with either PEG 200 (blue), PEG 300 (grey), or PEG 400 (violet) as the solvent. (**B**) ATR-IR spectra for membranes made from EPON and MBCHA with either PEG 200 (blue), PEG 300 (grey), or PEG 400 (violet) as the solvent. (**C**) Simple images of circular membrane cutouts: (**i**) EPON 200 membrane, (**ii**) BADGE 200, (**iii**) BADGE 300 with interfacially polymerized EPON active layer.

As illustrated in Figure 2A–C, a 7″ × 7″ × 1/8″ borosilicate glass sheet was coated with a thin layer of Slide Epoxease mold-release agent. An 11 cm × 14 cm sheet of 6 g/m^2^ carbon fiber veil was placed onto the coated glass and impregnated with the desired epoxy resin by directly pouring onto the fiber and allowing the resin to distribute itself undisturbed. The entire substrate was then placed into an oven set to 130 °C for 4 h. The cured composite was allowed to cool to room temperature and submerged overnight in a bath of deionized water to remove the PEG solvent. If the membrane did not separate from the borosilicate upon soaking in deionized water, the membrane was removed from the substrate by gently peeling by hand. Membranes made from EPON were transparent yellow in appearance regardless of the porogen (Figure 3C(i)). Membranes made from BADGE ranged from an opaque white color (Figure 3C(ii)) to a transparent blue color (Figure 3C(iii)) depending on the type of PEG used.

### 2.5. Interfacial Polymerization

Select membranes were cut into circular coupons and placed into a custom-made interfacial polymerization reactor such that the smooth side of the membrane (the side facing the glass plate during curing) faced the organic solution (Figure 2D). One side of the membrane was contacted to a 1–5% aqueous solution of tetramethyl N,N,N′,N′ tetramethyl-1,6 hexane diamine (TMHD). The organic layer consisted of a 1% solution of EPON resin in toluene. The reactor was allowed to sit for 4 h during which time the TMHD diffused from the organic layer to the aqueous layer, initiating the self-polymerization of the EPON monomer. After 4 h, the coupon was removed from the reactor and placed in a water bath for at least 24 h before use. The interfacial polymerization (IP) was visually apparent as the membrane took on an opaque yellow color.

### 2.6. SEM

Membrane samples were rendered conductive by sputtering with gold and imaged using a JEOL JSM-6700F FE-Scanning Electron Microscope (Jeol USA Inc., Peabody, MA, USA) or via Thermo Fisher Phenom Pharos G2 Desktop SEM FEG-SEM, sourced from Nanoscience Instruments (Alexandria, VA, USA) [35]. Fibers were imaged without sputtering via Nova NanoSEM 230 (FEI Company, Hillsboro, OR, USA, now ThermoFisher).

### 2.7. Membrane Testing

Membranes were tested for DI flux and solute rejection in a Sterlitech HP4750 stirred dead-end cell (Sterlitech, Auburn, WA, USA) pressurized with compressed nitrogen gas. The DI flux was measured gravimetrically. Crossflow testing was performed in a custom rig with a temperature-controlled DI (20–29 °C) and crossflow velocity of 10–15 cc/min. Flux was measured using a Tovatech FlowCal 5000 (Tovatech, Plano, TX, USA).

### 2.8. Dye Rejection

Rejection values were collected for and are reported as the average of 3 different coupons for each sample. After compacting with DI water at 50, 100, 200, 300, and 400 PSI until a steady flux was achieved, the DEC was loaded with 250 mL of 35 µM Rose Bengal (973.67 g/mol), 35 µM of Methylene Blue (319.85 g/mol), or 1 g/L of PEG 200 or 300. The cell was pressurized to 400 psi (27.6 bar). The first ~5 mL of permeate was discarded and the next 50 mL of permeate was collected for analysis. A calibration curve comprised of at least 5 standards was prepared to determine the concentration of dye permeates. Rejection was determined spectrophotometrically for dyes using Beer’s law, using total organic carbon analysis (TOC, Shimadzu TOC-LCSN) for PEG, and using conductivity for preliminary MgSO_4_ rejection.

### 2.9. XPS

XPS was performed using a Kratos Axis Ultra X-ray Photoelectron Spectrometer (Kratos Analytical, Manchester, UK). Samples were cleaned using Ar^+^ sputtering for 1 min prior to measurement. Data were processed using CasaXPS Version 2.3.23PR1.0 software package with CasaXPS R.S.F. libraries.

### 2.10. XRD

To compare pristine fibers with those extracted from the epoxy, XRD performed on a PANalytical X’Pert Pro X-ray Powder (Malvern Panalytical, Almelo, The Netherlands) Diffractometer was used to analyze any changes in crystallinity. Pristine EPON 300 (E300) membranes were cut and placed into excess commercial bleach with mild stirring. As a control, carbon fibers of the same area were placed into commercial bleach for the same time duration. Over time, the fibers agglomerated and the epoxy matrix separated into the solution as particulates and/or dissolved components. Fibers were washed with DI water and dried in a vacuum oven at 60 °C overnight before analysis.

### 2.11. Mechanical Testing

Tensile testing was performed using an Instron universal testing machine (Model 5944). Membranes were cut into 1 cm wide strips and 4 cm segments were tested at 0.1 mm/s.

### 2.12. Membrane Degradation and Fiber Recycling

The fact that hypochlorite was used to degrade epoxy membranes does not counteract claims that epoxies are tolerant to chlorine. The concentrations used for degradation are much higher than any concentration of chlorine added to water for disinfection purposes (~10,000× higher). Depending on the experiment, membranes were degraded using commercial bleach (8.25% sodium hypochlorite, 1.1 M) by either placing them in a still bath (to keep the veil structure intact) or by adding excess bleach and stirring using a magnetic stir bar for between 15 min and 3 h. For reuse of the veil for membrane fabrication, membranes were placed onto a glass plate and submerged in excess commercial bleach, then allowed to degrade overnight to completely remove the epoxy without disturbing the veil structure (Figure S7). The resulting fibers were then floated onto a glass substrate coated with Epoxease and gently rinsed with DI water. This veil was then used to make a new membrane.

## 3. Results and Discussion

### 3.1. Effects of Different PEG Mw during Synthesis

**Figure 4:**
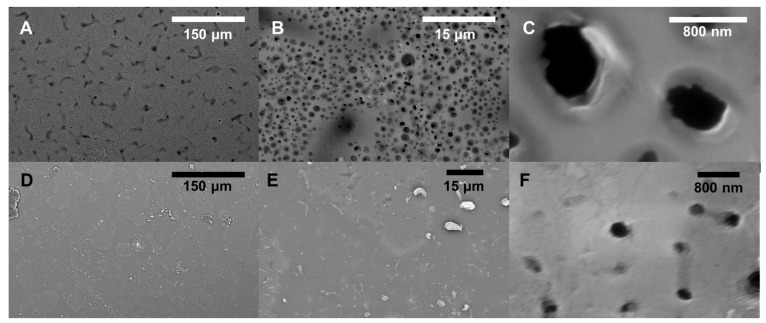
SEM images of sample epoxy surfaces at varying magnification: (**A**–**C**) B200, (**D**–**F**) B400.

**Figure 5:**
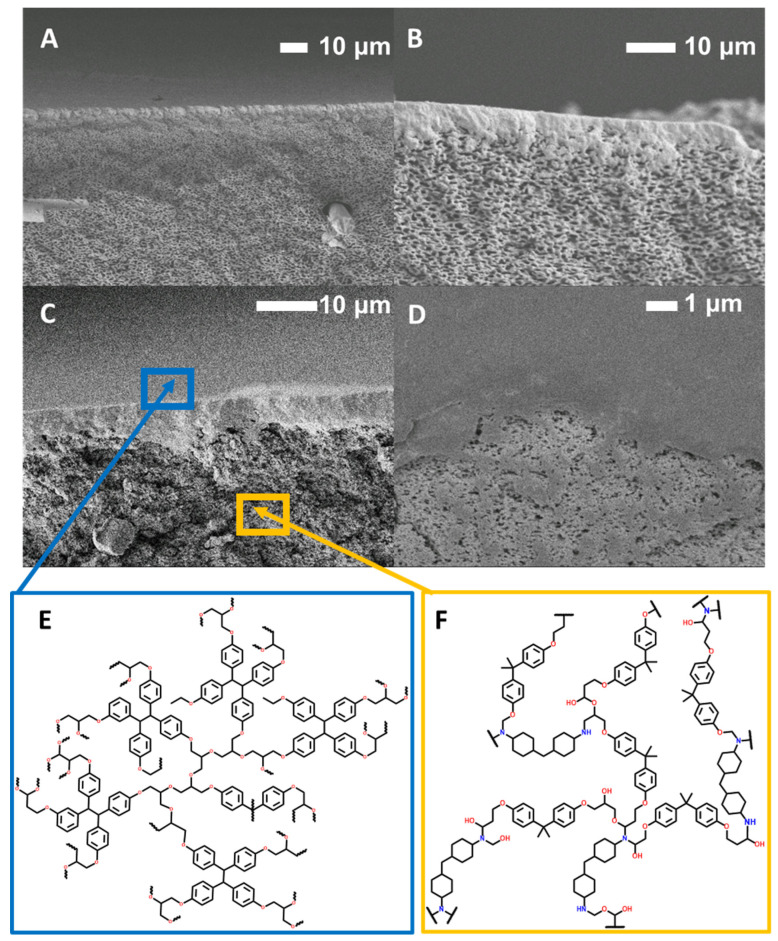
(**A**,**B**) SEM cross-sectional images of B200 with an interfacially polymerized EPON active layer. (**C**,**D**) SEM cross-sectional images of B300 with an interfacially polymerized EPON active layer. (**E**) An EPON active layer structure. (**F**) A BADGE-MBCHA support layer structure.

As demonstrated previously, rejection and flux can be tuned by changing the solvent/porogen system used in fabrication, where lower-molecular weight PEG leads to more porous films with lower rejection, while higher molecular weight PEG leads to denser structures with higher rejection [7]. The PEG acts as both a solvent to dissolve the epoxide and diamine components and as a pore-forming agent that is completely removed after curing. Without the PEG, the epoxy is completely nonporous. But, it also has a catalytic effect based dominantly on end-group concentration rather than molecular weight. If higher molecular weight PEG were to be used, this end-group effect may diminish, but that is beyond the scope of this work. Figure 4 includes SEM images of the BADGE-based membranes using different *M_w_* PEG in the synthesis where Figure 4A–C gives the most porous, made with PEG 200, and Figure 4D–F gives the least porous, made with PEG 400. The binary photos and calculated porosity from these images are included in Figure S4 and Table S2. This can also be observed in Figure 5, where supports were made using PEG 200 (Figure 5A,B) and PEG 400 (Figure 5D,E). The mechanical properties of the BADGE-based membranes are superior to the pure EPON membranes, as they are more flexible, even after drying out, while the EPON membranes, as expected for a more crosslinked network, are more brittle and become deformed when dried. While the chemical components have not changed, ATR-IR does reveal some of the nuances in functional groups that a more or less cured system would exhibit. As the MW of PEG is increased, the intensity of the O-H IR stretching, which is highlighted in yellow in Figure 3, increases, indicating that more C-N bonds have formed from ring opening. The regions likely to correspond to N-H bending (violet) and C-N bending show a subtle increase in the doublet intensity, which may be due to there being more crosslinking with the PEG 200 porogen (fewer primary and secondary amines, more tertiary amines), and thus a smaller and less variable N-H bend. These trends are the same regardless of the glycidyl ether (BADGE or EPON) used.

### 3.2. Performance and Characterization of BADGE-MBCHA Membranes

**Figure 6:**
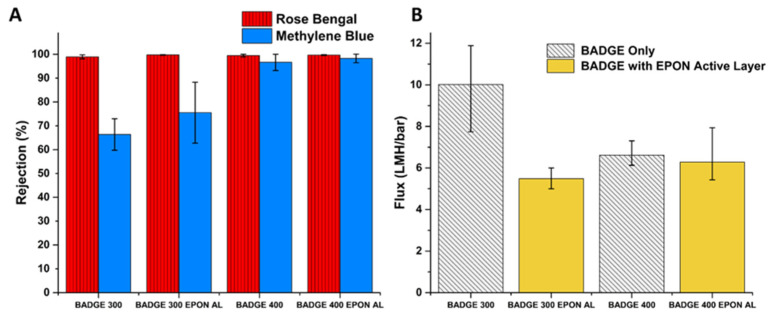
(**A**) Average rejection of Rose Bengal and Methylene Blue dyes with standard deviation for 3 membrane coupons. (**B**) Average DI flux measured in dead-end format with standard deviation for 3 membrane coupons.

**Figure 7:**
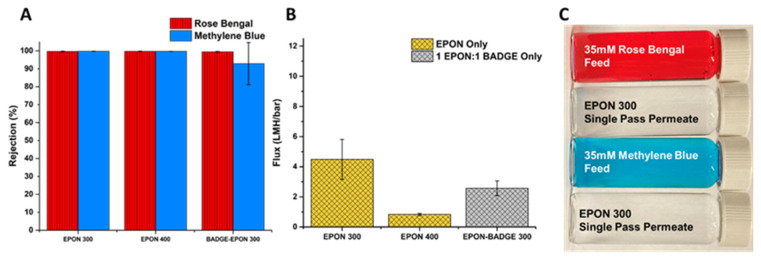
(**A**) Average rejection of Rose Bengal and Methylene Blue dyes with standard deviation for 3 membrane coupons. (**B**) Average DI flux through membranes measured in a dead-end format. (**C**) Images of feed and permeate solutions for an EPON 400 (E400) membrane with standard deviation for 3 membrane coupons.

Flux and rejection data for BADGE-based and EPON-based membranes are summarized in Figure 6 and Figure 7, respectively. The DI flux through the membranes ranged from 0.80 (Figure 7) to 10.0 LMH/bar (Figure 6), which is competitive with the solvent flux of commercial membranes with similar MWCO’s as well as highly novel membranes with much more complex syntheses/fabrication [38]. The membrane with the best permselectivity is the E300 membrane with an average DI flux of 4.48 LMH/bar and an average rejection of 99.6% and 99.8% for Rose Bengal and Methylene Blue dyes, respectively. The DI flux through the BADGE 300 (B300) membranes was significantly affected by the presence of the EPON active layer, dropping from an average of 10.01 to 4.58 LMH/bar. The rejection increased from 99.0% to 99.8% and from 66.4% to 75.5% for Rose Bengal and Methylene Blue, respectively. However, the standard deviation in rejection increased significantly for the B300 with an EPON active layer, indicating less consistent quality of the barrier layer. Although further optimization of the flux was not the purpose of this project, bisphenol A propoxylate diglycidyl ether can be substituted to further increase flux.

The decrease in porosity from changing the support formulation to PEG 400 rather than using PEG 300 as the porogen caused the flux to decrease from an average of 10.01 to 6.61 LMH/bar (Figure 6B). The further addition of the EPON active layer resulted in a drop of just under 0.30 LMH/bar, although, as with the B300 membranes, the B400 membrane showed greater variability in flux (higher standard deviation) with an active layer than without an active layer. The drastic increase in Methylene Blue rejection from 66.4% for B300 to 96.6% for the B400 membrane demonstrates that the porosity of the support has a greater effect on the rejection of the membrane than the addition of an active layer under these synthesis conditions (Figure 6A). Still, the EPON active layer increased the rejection of Methylene Blue from 96.6% to 98.3% and small enhancements such as this may be industrially significant.

When it comes to IP for traditional PA membranes, generally, smaller pore sizes lead to a more uniform and thicker active layers as the diffusion of MPD into the organic TMC layer is slower and more controlled [35,36,37]. With larger pores, the diffusion is less controlled and the movement of MPD more rapid, resulting in a rougher active layer as well as polymerization occurring in the pores. There tends to be less crosslinking due to PA layers forming inside the pores, increasing the diffusion path of MPD to reach the organic phase [39]. In addition to pore size, the number of pores per unit area affects the active layer morphology and thickness of an IP active layer [35]. Generally, higher surface porosity enables continuous film formation with higher crosslinking density when compared to membranes with support layers with lower surface porosity [35,36,37]. Tracking with this, highly porous membranes prepared using epoxy cured in PEG 200 did not result in significant rejection of either Rose Bengal (RB) or Methylene Blue (MB), even with the presence of the EPON active layer. Given the volumetric limits of the dead-end cell system used, the flux was too high to measure. Still, SEM images of these membranes show that a continuous active layer on the surface of the support was formed, with the active layer protruding into the porous layer of the support (Figure 5A,B). In contrast, the active layer of the B300 membrane appears to be sitting on top of the support layer (Figure 5D) [41]. Taking a closer look at the active layer (Figure 5E) of the B300 membrane where the active layer has been peeled back, the smaller pores of the B300 support are revealed.

### 3.3. Performance and Characterization of EPON-MBCHA Membranes

Dramatic increases in rejection can be achieved without the addition of an active layer. To further improve selectivity of BADGE membranes, one option would be to use a PEG of higher molecular weight to see if the trend of decreasing pore size continues. But higher molecular weight PEGs below *M_w_* 1000 are less common, and at the range of 1000+ *M_w_*, PEGs melt at temperatures above room temperature, introducing new variables into the synthesis of epoxies when compared with lower MW PEGs. Replacing the bi-functional BADGE with the tetra-functional EPON (Figure S1) gave a new type of epoxy matrix. As expected and as discussed in the Introduction, the flux through the membranes was greatly reduced, but was still tunable by changing the *M_w_* of the porogen, as with the BADGE membranes. Changing the porogen from PEG 300 to PEG 400 showed a more than five-fold reduction in flux, dropping from an average of 4.48 to 0.84 LMH/bar (Figure 7B). Surprisingly, and despite the fact that the EPON-based epoxies are intrinsically more crosslinked, those made with PEG 200 as the porogen showed extremely high water permeability and negligible rejection of dyes. The flux data reveal that the EPON-MBCHA system is more sensitive to the effects of the change in porogen when compared to the BADGE-MBCHA membranes.

Interfacial polymerization was attempted, but only successful for BADGE-MBCHA supported membranes. When EPON-MBCHA membranes were used, the EPON layer was not well adhered to the support, and it was easily damaged on handling or with any shear force applied to the membrane. This is likely due to the EPON-MBCHA membranes being too dense, such that the diffusion of the TMHD was hindered, and thus only a very thin film at the very surface of the support formed. Figure S4 shows a cross-sectional image of an E300 support membrane with an interfacially polymerized active layer. Compared to the membranes in Figure 5, its active layer is much thinner, on the scale of hundreds of nanometers.

**Figure 8:**
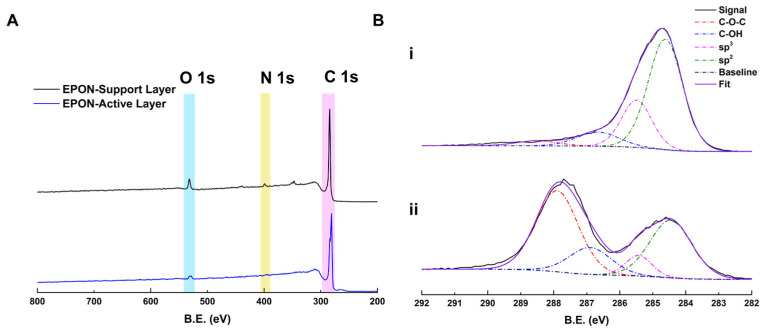
(**A**) Survey spectra for an E400 membrane with an interfacially polymerized EPON active layer. Support side shown in black (top) and active layer side shown in blue (bottom). O1s, N1s, and C1s regions highlighted in blue, yellow, and pink respectively. (**B**) Hi-resolution scans of the C1s region of the XPS spectra deconvoluted. (**i**) EPON-MBCHA Support Layer; (**ii**) EPON active layer.

Although performance results were not obtainable, the EPON-MBCHA membrane with an IP EPON active layer was analyzed via XPS (Figure 8) to determine elemental composition and observe the C1s region. As expected, the EPON active layer does not have any nitrogen present (region highlighted in yellow), while the support layer does, due to the presence of the MBCHA monomer. Deconvolution of the C1s region suggests that, compared to the E300 layer, the EPON active layer has a large amount of unreacted epoxide groups after interfacial polymerization with a large peak at 288.5–287.9 eV assigned to C-O-C groups. While this may be due to the shallow depth of analysis for XPS and the fact that the layer observed was the organic-facing side, to achieve better separation and/or adhesion to the support, additional exposure to TMHD (or another tertiary amine) after the active layer is grown may be required to achieve a denser, and thus effective, active layer. That way, any unreacted epoxides would participate in crosslinking rather than continuing to react with additional EPON in solution.

Although there were drastic changes in flux dependent on the PEG used during polymerization of the EPON-MBCHA membranes, the differences in rejection of the dyes were much less pronounced. Both the E300 and E400 membranes achieved average rejections of Rose Bengal and Methylene Blue above 99.7% (Figure 7A). Thus, the E300 membrane is the best option of the fabricated membranes for aqueous separations observed in this work.

In an attempt to balance the high flux and superior mechanical properties of the BADGE membranes with the high rejection of the EPON membranes, membranes were made with a 1:1 combination of BADGE and EPON with MBCHA. Despite being synthesized in PEG 300, this membrane’s DI flux was lower than that of the E300 membranes and the rejection of Methylene Blue was less consistent (Figure 7A,B).

To assist in determining the mechanism of rejection, rejection of PEG 200 and 300, which have MW values similar to Methylene Blue was tested with a feed concentration of 1 g/L. According to zeta potential measurements (Figure S6A), the EPON membranes are charged; hence, charge exclusion could play an important role in rejection of feed solutes. As expected, rejection of PEG 200 and 300 was limited, between 5 and 30% (Figure S6B). There is also the possibility that these solutes are poor choices for rejection of uncharged organics, simply because the membranes themselves are cured using PEG as a solvent, meaning leaching of any residual PEG into the permeate could cause the measured rejection to be lower. Despite being charged membranes, rejection of MgSO_4_ was measured to be between 20% and 30%. Therefore, the membranes are useful for separating out charged organic dyes, but not uncharged organics or inorganic ions.

### 3.4. Chemical and Physical Degradation of Epoxy Membranes for Recycling

Mechanical means or retrieving carbon fibers from highly crosslinked networks usually leads to some loss of the graphitic nature or length of individual fibers. Another way of retrieving them would be by applying chemical treatment to systematically destroy the matrix and not the fibers. In this work, we have managed to non-destructively retrieve the fibers from the composite membranes. Degradation of the epoxy matrix to retrieve the fibers was achieved by treating membranes with commercial bleach for 1 h for simple removal to 24 h for completely removing the epoxy, while keeping the veil structure intact. While not measured in a controlled manner, the timeline for degradation was shorter for membranes that had been used in separations, likely due to some mechanical wear or removal of any unreacted monomeric materials and PEG, allowing better chemical infiltration compared to pristine membranes.

**Figure 9:**
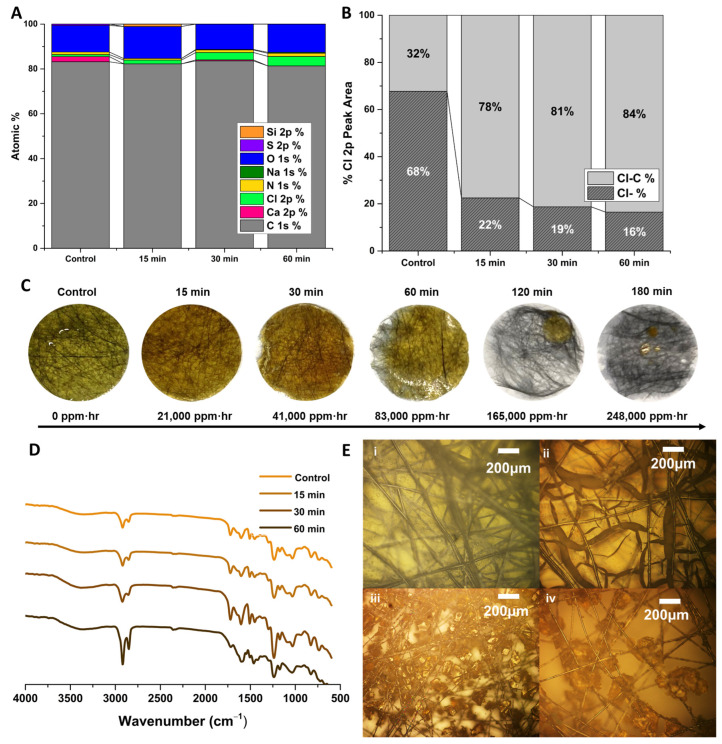
EPON-MBCHA membrane polymerized in PEG 400 (E400). (**A**) Summary of survey spectra for pristine E400 and E400 after exposure to hypochlorite for 15, 30, and 60 min. (**B**) Summary of peak-model components for the Cl 2p region of the XPS spectra (**C**) Images of membrane samples with exposure to hypochlorite with time converted to ppm·hr. (**D**) ATR-IR spectra for pristine and degraded samples. (**E**) (**i**) Dried E400 membrane. E400 membrane after exposure to hypochlorite for (**ii**) 15, (**iii**) 30, and (**iv**) 60 min.

**Figure 10:**
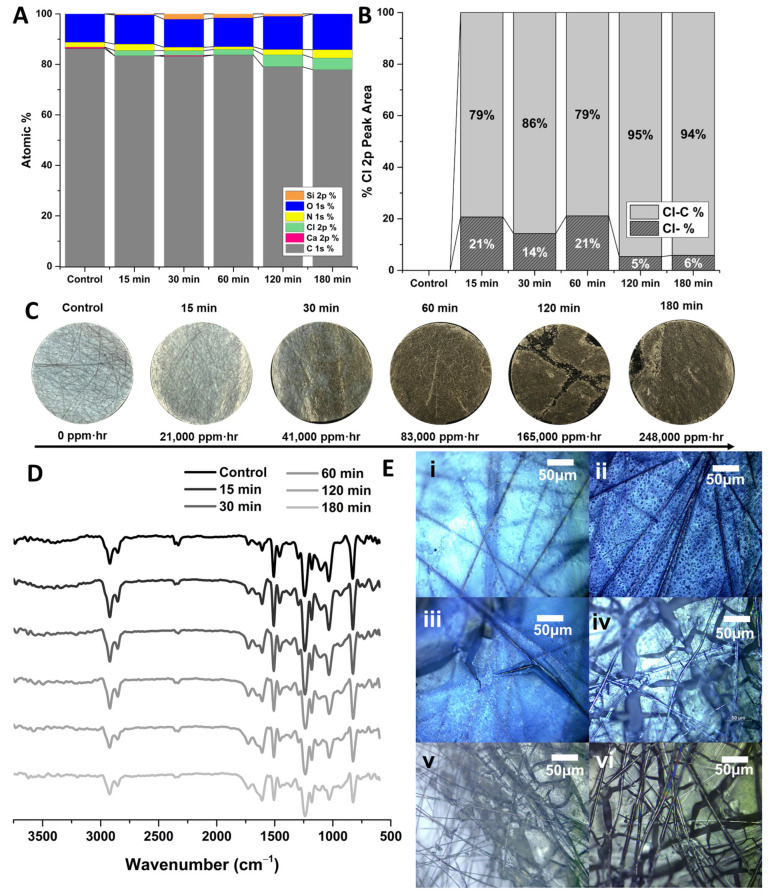
BADGE-MBCHA membrane polymerized in PEG 300 (B300). (**A**) Summary of survey spectra for pristine B300 and B300 membranes after exposure to hypochlorite for 15, 30, and 60 min. (**B**) Summary of peak-model components for the Cl 2p region of the XPS spectra. (**C**) Images of membrane samples with exposure to hypochlorite with time converted to ppm·hr (concentration of solute in ppm multiplied by the exposure time in hours). (**D**) ATR-IR spectra for pristine and degraded samples. (**E**) (**i**) Dried B300 membrane. E400 membrane after exposure to hypochlorite for (**ii**) 15, (**iii**) 30, (**iv**) 60, (**v**) 120, and (**vi**) 180 min.

It was initially presumed that the degradation of the membrane was chemical in nature. The XPS data of the degraded polymers in Figure 9A and Figure 10A show the elemental analysis of the polymers with increasing exposure to concentrated hypochlorite. The elemental analyses show an increase in the amount of chlorine present in the E400 samples as they degrade. The B300 samples appear to have a similar increase in chlorine present as well as an increase in oxygen (Figure 10A). A peak model was developed to deconvolute the Cl 2p region of the XPS spectra into ionic Cl (Cl^−^) and organic-bound Cl (C-Cl in the legend). These data are summarized in Figure 9B and Figure 10B showing a change in the nature of the Cl in the samples with prolonged exposure, with a slight increase in the C-Cl and the reduction of Cl^−^ over time. This could be due to chlorination of the polymer by free chlorine and a reduction of surface-bound hypochlorite, although the peak model alone cannot determine this. Representative peak fits for these data can be found for the E400 samples in Figure S7. Analysis of the C1s region of the XPS spectra (Figure S7) indicates some oxidation of the polymer with exposure to hypochlorite, with an increase in the C-O, C=O, C-O-C, and C-Cl components of the peak model with time. It is difficult to truly differentiate between reduction of O-H groups and chlorination, although the peak model suggests both.

Although there are likely some chemical changes due to hypochlorite exposure, other data suggest a mechanical mechanism. Other works employ the use of acids or supercritical water in attempts to chemically recycle epoxy resins [42,43]. Previous studies had determined that BADGE-MBCHA membranes were somewhat stable in aqueous hypochlorite, while it was anecdotally known that the membranes would completely disintegrate/dissolve if left overnight in 8.25% hypochlorite. Since this concentration is so much higher than that needed industrially for disinfection, this is not of major concern regarding our claims to improved chemical tolerance. Still, chemical analysis, specifically of the E400-MBCHA membranes, suggest a different mechanism. Hypochlorite can oxidize primary and secondary alcohols to carbonyls among many others, of which some must lead to chain scission within the epoxy either directly or by cascade. Still, based on work studying the degradation of epoxy in concentrated acid, it is likely that the mechanism is also due to restricted electrolyte diffusion [44]. The intense swelling that occurs as ionic species propagate through the samples causes fracturing of the network as it unevenly expands. This is supported by the IR spectra in Figure 9D and Figure 10D, where only minor changes to the IR spectra are observed. Figure 9D indicates a minor increase in the relative strength of the O-H stretch, but no significant changes in functional groups that would imply intensive chemical degradation. Figure 10D indicates even less of a change as the BADGE samples degraded. Figure 9E and Figure 10E are optical microscope images of E400 and B300 samples, respectively, before any exposure to hypochlorite. With prolonged exposure to hypochlorite, the polymer appears to fracture into smaller and smaller pieces, physically separating from the fibers. The E400 samples fractured into larger pieces and separated from the fibers more quickly. This is likely due to the differences in the crosslink density and polarity of the network. The higher crosslink density of the EPON-based samples fractured into larger pieces and separated from the fibers more quickly (Figure 9C) than the BADGE membranes, which degraded into a fine solid (Figure 10C).

**Figure 11:**
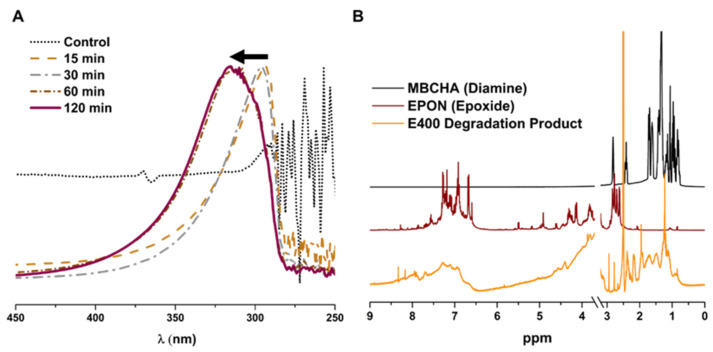
(**A**) UV-vis of water-soluble portion of degraded polymer. Black arrow indicates shift to longer wavelength over time. (**B**) NMR spectra for MBCHA, EPON, and the DMSO-soluble portion of the E400 degradation product.

UV-vis spectra were collected for the aqueous component of the degraded polymer. After the membranes soaked in hypochlorite, the degraded membranes were added to DI water and UV-vis of the solution was measured (Figure 11A). The absorbance of the supernatant for membranes soaked for longer than 30 min showed a significant red-shift. NMR analysis of the monomer components, EPON and MBCHA, and the degraded polymer (soluble portion in d-DMSO) shows substantially broader peaks, indicating that the product is still polymeric/oligomeric. While additional analyses would be required to fully understand the degradation mechanism for the samples, this is beyond the scope of this work.

### 3.5. Evaluation of Recovered Carbon Fibers

**Figure 12:**
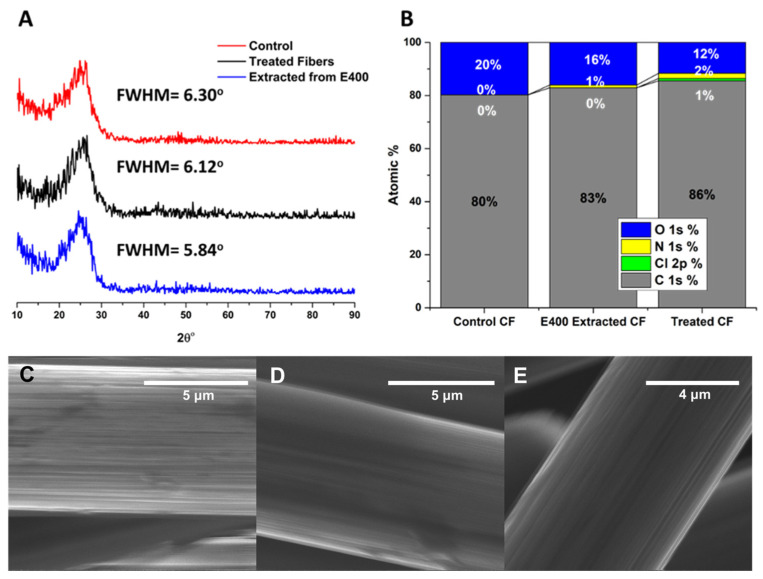
(**A**) XRD spectra and FWHM for graphitic peak at 25° 2θ for a pristine CF veil, a CF veil treated with hypochlorite solution, and a CF veil extracted from an epoxy matrix. (**B**) XPS derived elemental analysis for the fibers in A. (**C**–**E**) SEM images of individual CF fibers in pristine veil, CF veil treated with hypochlorite, and CF veil extracted from an epoxy matrix.

**Figure 13:**
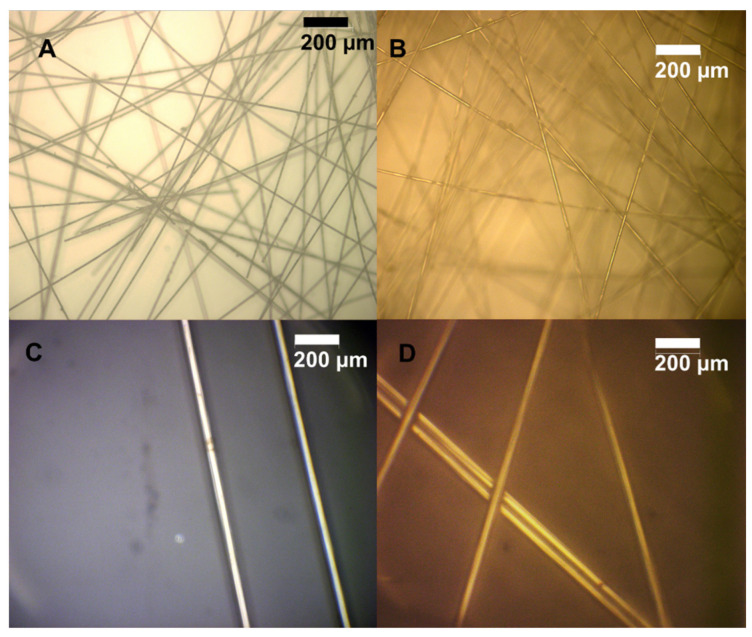
(**A**) Optical microscope images of pristine fibers at 10× magnification. (**B**) Optical microscope image of fibers extracted from E400 resin after 60 min. (**C**) Pristine fibers at 100× magnification. (**D**) Extracted fibers at 100× magnification.

The carbon fibers used in this work were derived from polyacrylonitrile (PAN), which, upon exposure to heat, forms the graphite-like ribbons that comprise carbon fibers. The quality of the recovered carbon fibers was evaluated using XRD, XPS, optical microscopy, and tensile strength measurements. Pristine carbon fibers were compared to fibers that had been treated with hypochlorite (as a control) and fibers extracted from the E400 resin. XRD reveals a distinct peak at 25° 2θ, indicative of the crystalline graphite peak (Figure 12A). A simple gaussian fit to the XPS data using OriginPro peak fit indicates that there was no substantial difference in the location or FWHM of the peak, which was 6.30° for pristine fibers and 5.84° for those extracted from the epoxy matrix. XPS analysis shows a decrease in oxygen content when comparing pristine fibers to extracted fibers to pristine fibers treated with hypochlorite. The treated fibers, not extracted from epoxy, showed a small amount of chlorine (1%) as derived from the XPS survey spectra (Figure 12B). This could be due to some chemical modification that occurs when fibers are exposed to hypochlorite without the protection of the epoxy layer, or could be some physisorption that is simply not detected in the other samples. SEM images of the surfaces of carbon fibers show no difference between either control and the “recycled” fibers (Figure 12C–E). Optical microscopy images of the recycled fibers were indistinguishable from the pristine fibers. No substantial differences in fiber diameters (~15–26 μm) or shape were observed (Figure 13). All of these data suggest that there were likely no substantial changes to the mechanical properties of the fibers.

**Figure 14:**
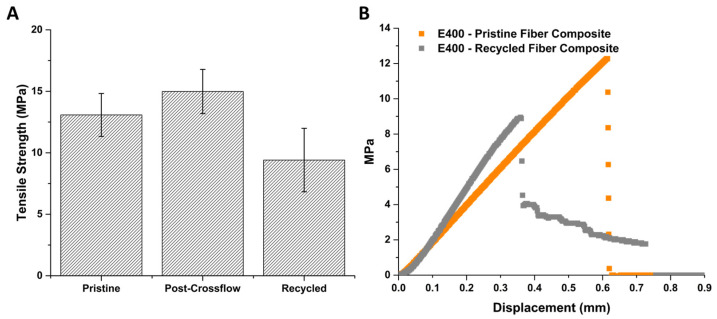
(**A**) Tensile strength of E400 composite membranes. (**B**) Stress–strain plot for representative samples showing catastrophic vs. gradual failure.

To support this, measurement of the tensile strength of individual carbon fibers suspended between pieces of epoxy was attempted, but proved impossible given available instrumentation and the fragility of nonwoven fibers compared to woven strands. Therefore, the mechanical integrity of the fibers was indirectly evaluated by comparing pristine E400 composites to E400 composites measured after exposing them to crossflow pressure, to those remade from fibers extracted from E400 resin. Figure 14A summarizes the yield strength of the different composites suggesting a decrease in the strength of the composite when using recycled fibers. Application of pressure via crossflow shows no decrease in the mechanical integrity of the samples over the short timescale tested even though some compaction occurred (Figure S9). Still, as mentioned in the procedure section, the method for re-making the membrane is not ideal and the fiber spacing and orientation is inevitably compromised upon transfer (in water) from one glass substrate despite extreme care. This is also supported by comparing the failure mechanisms for the different composites. All of the pristine composites and the post-crossflow samples exhibited catastrophic failure. On the other hand, some samples made from the recycled fibers showed partial failure at an initially lower applied force, but samples then underwent additional elongation before final breakage (Figure 14B). Composites, when uniform, exhibit physical properties that are the weighted average of the individual components. It is likely that the quality of the lab-scale recycled composites has a lack of uniformity where in a real recycling setting, fibers could be re-dispersed appropriately before being incorporated into a new polymer matrix. This, along with the minimal difference in tensile strength between the composites with recycled fibers and those made with pristine fibers, is likely an indicator that the integrity of the fibers is, in fact, maintained.

### 3.6. “Recycled” CF Composite Membrane Performance

**Figure 15:**
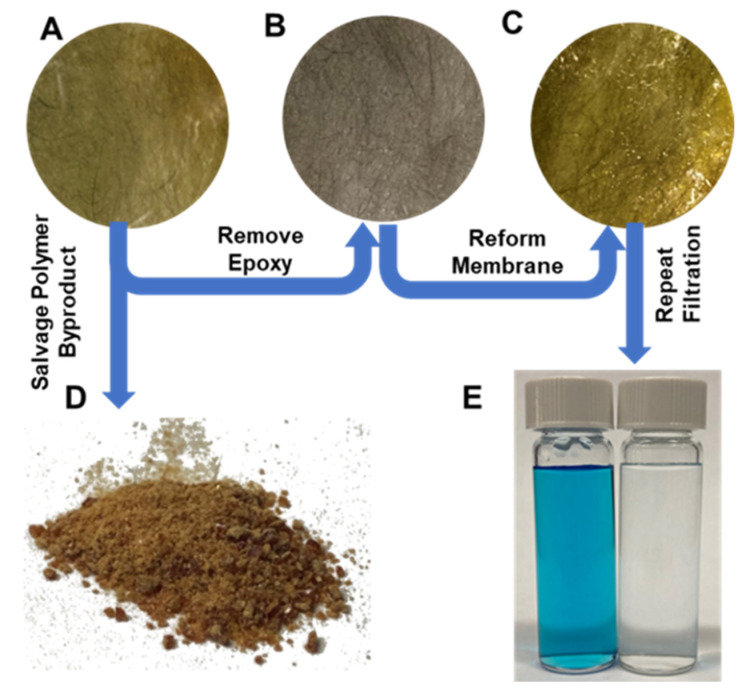
(**A**) EPON-MBCHA membrane (E400). (**B**) Carbon fiber veil extracted from A. (**C**) E400 membrane fabricated using a fiber veil from (**B**). (**D**) Polymer powder extracted from E400 membrane. (**E**) Image of Methylene Blue feed (left) and permeate (right) after cleaning using the membrane in (**C**).

The process for recycling the fibers for reuse in a new membrane was described earlier in this text and summarized in Figure 15. Figure S9 provides full images of an E400 membrane, the extracted veil, and the re-formed membrane. While there was less uniformity in the veil’s fibers, the membrane still performed well under the identical testing conditions described previously. The resultant membrane was tested for rejection of 35 µM of Methylene Blue (the smaller of the two dyes tested) at 20% recovery in a dead-end cell. Rejection was above 99%, indicating that the recycled fibers do not compromise the performance of the membrane.

## 4. Conclusions

Presented here are seven different membrane formulations and the first that are purely amine–epoxy in nature as well as an epoxy active layer on top of an amine–epoxy supporting membrane. Nanofiltration of small-molecule dyes may be achieved without a separately-formulated active layer apart from any intrinsic asymmetry arising from the thermally cured films. Membranes fabricated using tetrafunctional EPON monomer with MBCHA diamine exhibited Rose Bengal and Methylene Blue rejection of greater than 99%. The addition of an EPON polyether epoxide active layer onto a more porous supporting layer increases rejection, but is not necessary for high rejection of small-molecule dyes. Replacement of polyamide membranes with polyepoxyethers may not yet be viable for applications like seawater desalination, but the benefits of this chemistry, including their chemical stability, manufacturing versatility, and ability to be optimized, make them valuable contributors to the water filtration toolbox.

Carbon fibers can be non-destructively extracted from the epoxy resins and reused without compromising filtration. Without the limitations of the lab-scale recycling process, in particular the re-formation of the veil, it is likely that recycled composites would be indistinguishable from those made with pristine fibers. Analysis of the epoxy resin byproduct shows minimal change from the original bulk polymer suggesting a mechanical-type erosion due to swelling by hypochlorite solution, while analysis of the byproduct that is water-soluble suggests more significant chemical changes that occur after 30 min of exposure to hypochlorite. These results suggest that the recycling of carbon fibers is achievable and may help make carbon fibers more relevant for a wider variety of products, especially if cheaper recycled fibers may be transferred from higher value products to lower-value/consumable products like membranes. The resin, although highly crosslinked, may therefore retain its mechanical properties and be used as a filler or converted to some other resource.

## Data Availability

The original contributions presented in the study are included in the article and Supplementary Materials, further inquiries can be directed to the corresponding author.

## Supplementary Materials

The following supporting information can be downloaded at: https://www.mdpi.com/article/10.3390/polym16182569/s1, Figure S1: Polymerization of EPON with MBCHA, Table S1: Summary of membrane formulations, Figure S2: SEM surface images of EPON membranes, Figure S3: SEM cross-sectional images of EPON Membranes, Figure S4: SEM cross-section of an E300 membrane with an EPON active layer, Figure S5: SEM Images converted to binary for porosity calculations, Table S2: Porosity and average pore size calculated from images in Figure S5, Figure S6: Zeta Potential and PEG Rejection, Table S3: Elemental analysis for degraded membranes, Figure S7: Cl2p and C1s Spectra for E400 membranes, Figure S8: Images of Membrane, Extracted Veil, and “Re-Made” Membrane, Figure S9: Crossflow Compaction Curve for E400 without Feed Spacer.
