# Supplementary material for: Monolithic Polyepoxide Membranes for Nanofiltration Applications and Sustainable Membrane Manufacture
Source: Polymers (Basel). 2024 Sep 11;16(18):2569. doi: 10.3390/polym16182569 (PMC11435267; doi:10.3390/polym16182569)
Supplement: Supplementary file 1 [file polymers-16-02569-s001.zip › polymers-3165233-supplementary.pdf]

Supplemental Information to Accompany Monolithic Polyepoxide Membranes for Nanofiltration Applications and Sustainable Membrane Manufacture

Mackenzie B. Anderson,[†] Riley A. Danna,[†] Clayton French,[†] Jishan Wu,[*] Markus N. Thiel,[†] Zhiyin Yang,[†] Eric M.V. Hoek[*,#] and Richard B. Kaner[†,‡]

[‡]Department of Chemistry and Biochemistry, [‡]Department of Materials Science and Engineering, and *Department of Civil & Environmental Engineering, University of California, Los Angeles (UCLA), Los Angeles, California 90095, USA
[#]Energy Storage & Distributed Resources Division, Lawrence Berkeley National Lab, Berkeley, California 94720, USA

Table S1: Summary of membrane formulations.

Name	Epoxide	Diamine	Solvent/Porogen	IP Active Layer (y/n)
**BADGE-200 (B200)**	1.0g BADGE	330 mg MBCHA	3.25g PEG 200	n
**BADGE-300 (B300)**	1.0g BADGE	330 mg MBCHA	3.25g PEG 300	n
**BADGE-400 (B400)**	1.0g BADGE	330 mg MBCHA	3.25g PEG 400	n
**EPON-200 (E200)**	1.0g EPON	330 mg MBCHA	3.25g PEG 200	n
**EPON-300 (E300)**	1.0g EPON	330 mg MBCHA	3.25g PEG 300	n
**EPON-400 (E400)**	1.0g EPON	330 mg MBCHA	3.25g PEG 400	n
**EPON-BADGE-300**	0.5g BADGE, 0.5g EPON	330 mg MBCHA	3.25g PEG 300	n
**BADGE-300-EPON AL**	1.0g BADGE	330 mg MBCHA	3.25g PEG 200	y
**BADGE-400-EPON AL**	1.0g BADGE	330 mg MBCHA	3.25g PEG 400	y
**EPON-300-EPON AL**	1.0g EPON	330 mg MBCHA	3.25g PEG 300	y

Table S2: Porosity and average pore size calculated from images in **Figure S5**.

Sample	Aerial Porosity (%)	Average Pore Diameter (μm)	Pore Diameter Standard Deviation (μm)
**B200**	19.8	1.36	0.59
**B400**	4.5	0.30	0.16

Table S3: Elemental analysis for E400 membranes with degradation.

Exposure Time (min)	Exposure (x1000 ppm·hr)	C 1s %	Ca 2p %	Cl 2p %	N 1s %	Na 1s %	O 1s %	S 2p %	Si 2p %
0	0	83.2	2.3	0.9	1.1	0.0	11.9	0.6	0.0
15	21	82.3	0.0	1.7	0.7	0.0	14.3	0.0	1.1
30	41	83.6	0.4	3.3	1.1	0.2	11.3	0.0	0.0
60	83	81.4	0.0	4.2	1.4	0.4	12.6	0.0	0.0

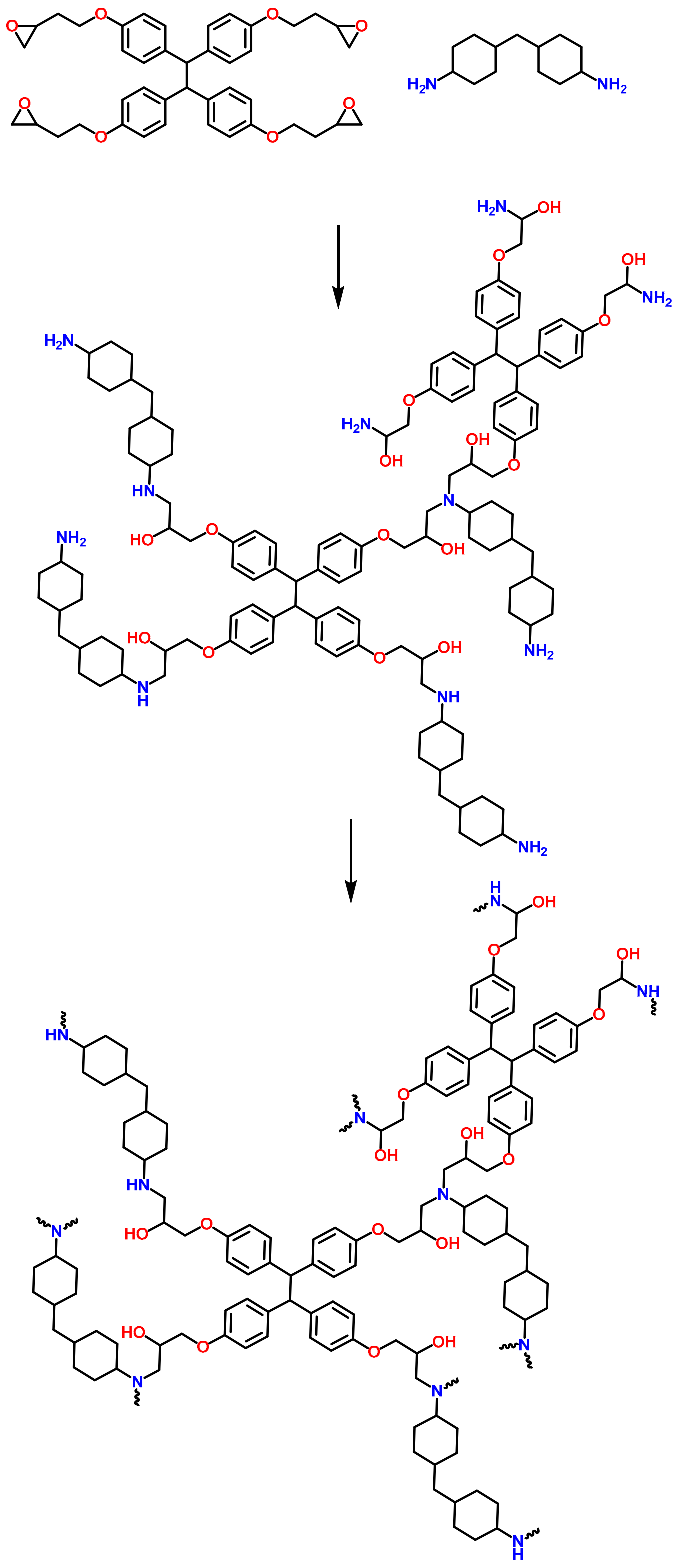

Figure SI: Polymerization of EPON with MBCHA.

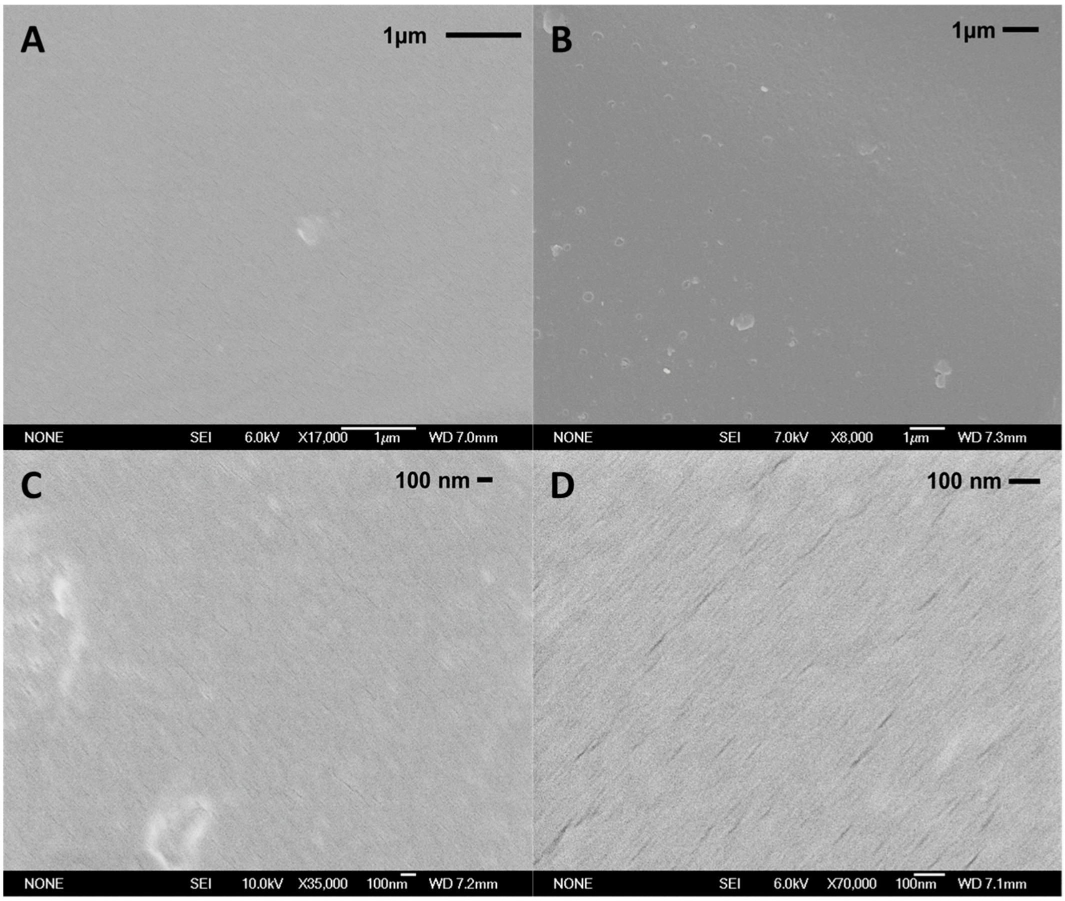

Figure S2: SEM surface images of EPON 300 membranes (A) and (C) and EPON 400 membranes (B) and (D).

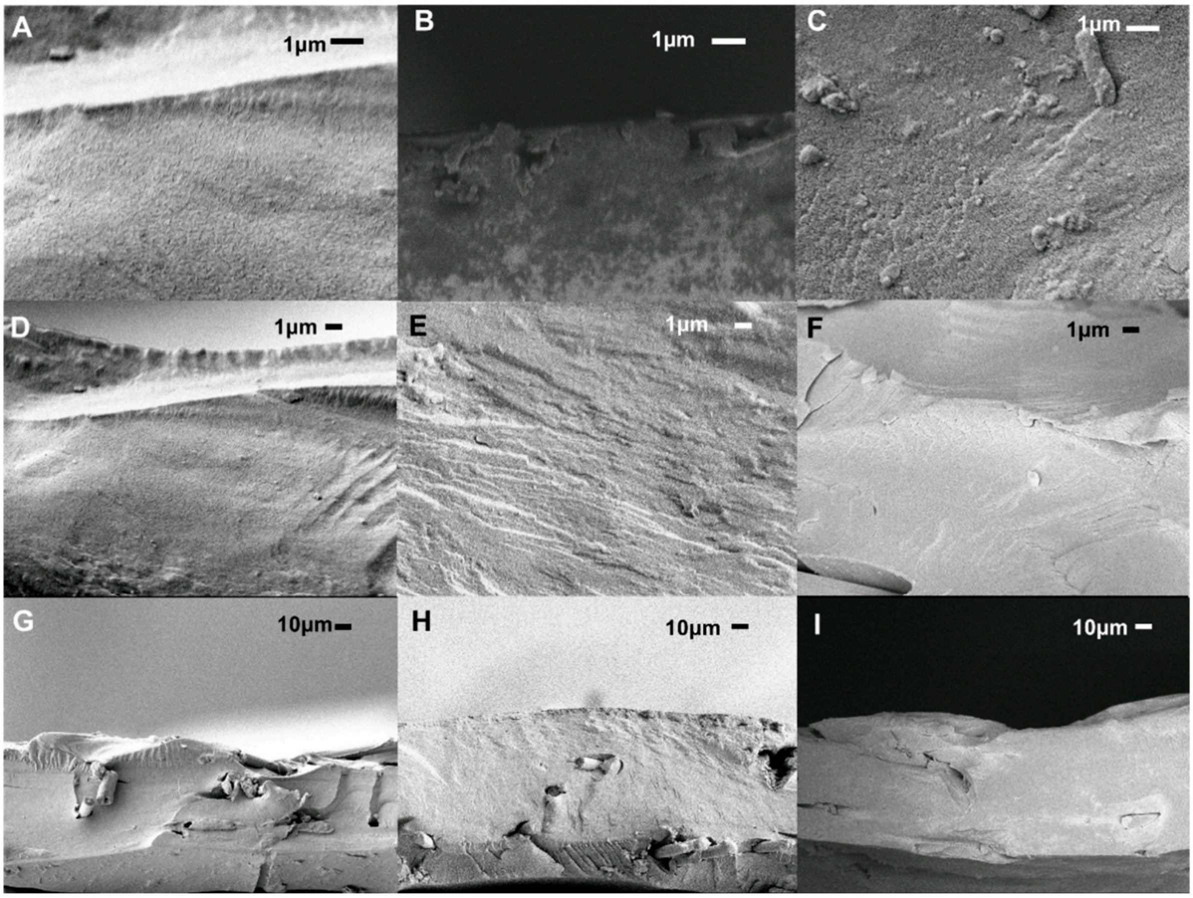

Figure S3: (A) SEM cross-sectional images of an EPON-MBCHA membrane made with PEG-300. (B) SEM image of an EPON-MBCHA membrane made with PEG-400 near the top (substrate-facing) surface. (C) Membrane made from a combination of BADGE and EPON epoxides and MBCHA. (D) Cross section of an EPON-MBCHA-PEG 300 membrane near the top of the membrane. (E) Cross section of an EPON-MBCHA-PEG400 membrane at reduced magnification. (F) Cross-section of an EPON-BADGE-MBCHA-PEG300 at reduced magnification. (G)-(H) SEM images of full cross-sections of EPON-MBCHA-PEG 300, EPON-MBCHA-PEG 400, and EPON-BADGE-MBCHA-PEG 300, respectively.

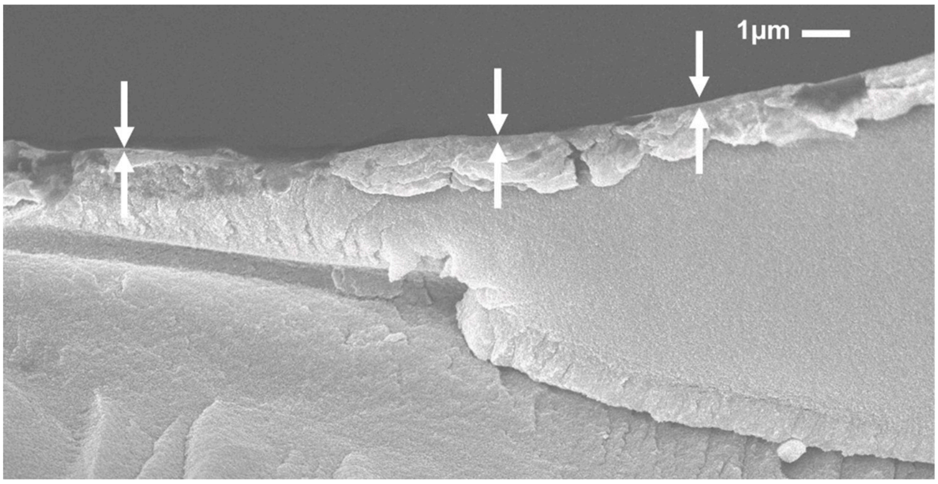

Figure S4: SEM cross-section of an EPON-300 membrane with an EPON active layer.

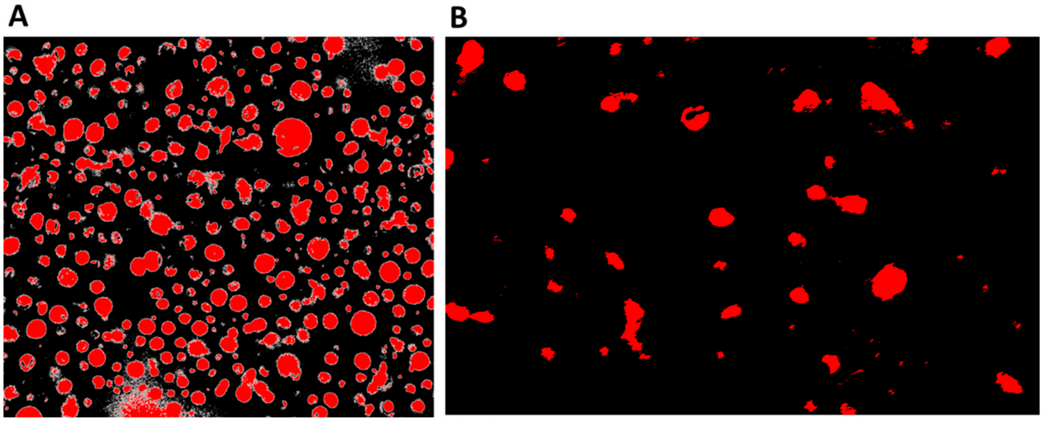

Figure S5: SEM Images converted to binary for porosity calculations **(A)** BADGE 200 **(B)** BADGE 400

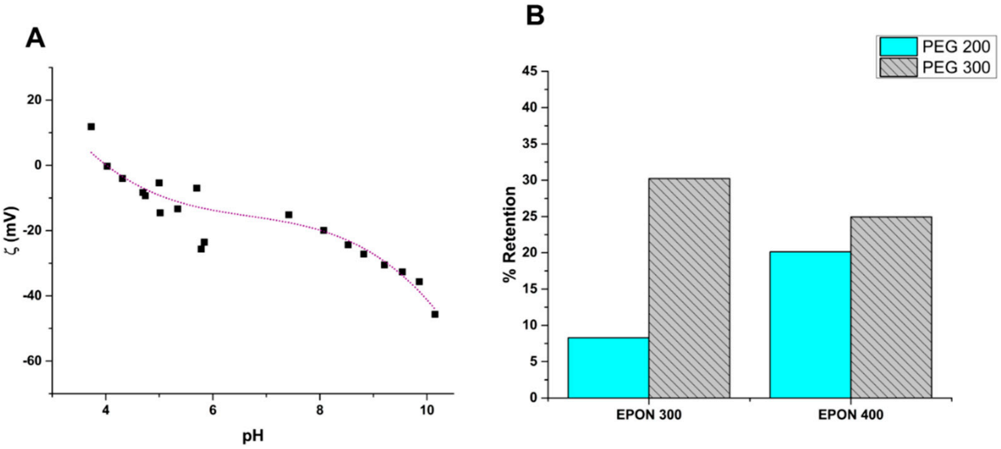

Figure S6: (A) Zeta Potential as a function of pH for EPON 400 (B) Rejection of PEG 200 and PEG 300 by EPON membranes.

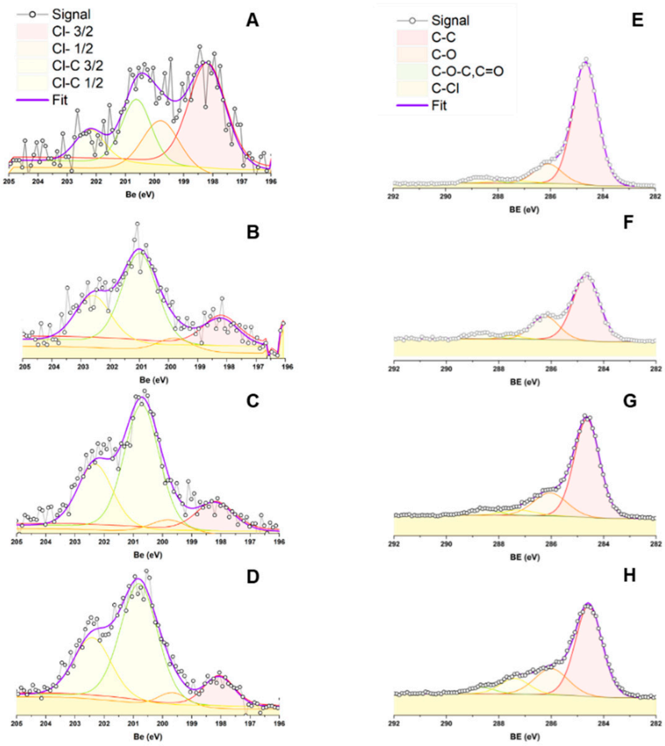

Figure S7: Cl 2p spectra for E400 membranes treated with hypochlorite **(A)** Control **(B)** 15 min **(C)** 30 min **(D)** 60 min C 1s spectra for E400 membranes treated with hypochlorite **(E)** Control **(F)** 15 min **(G)** 30 min **(H)** 60 min

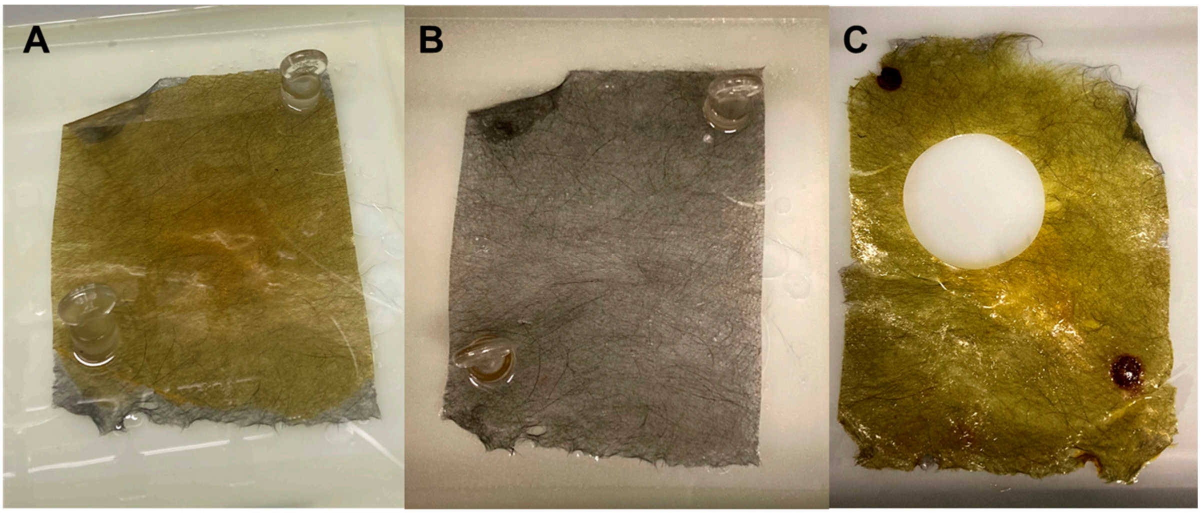

Figure S8: (A) E400 membrane submerged in hypochlorite solution, glass stoppers ensure sample remains submerged **(B)** E400 sample after 24hr, only CF veil remains **(C)** E400 membrane made from veil in **(B)** with sample coupon removed. Dark spots show where stoppers prevented polymer exposure to hypochlorite, therefore there is 2x the epoxy.

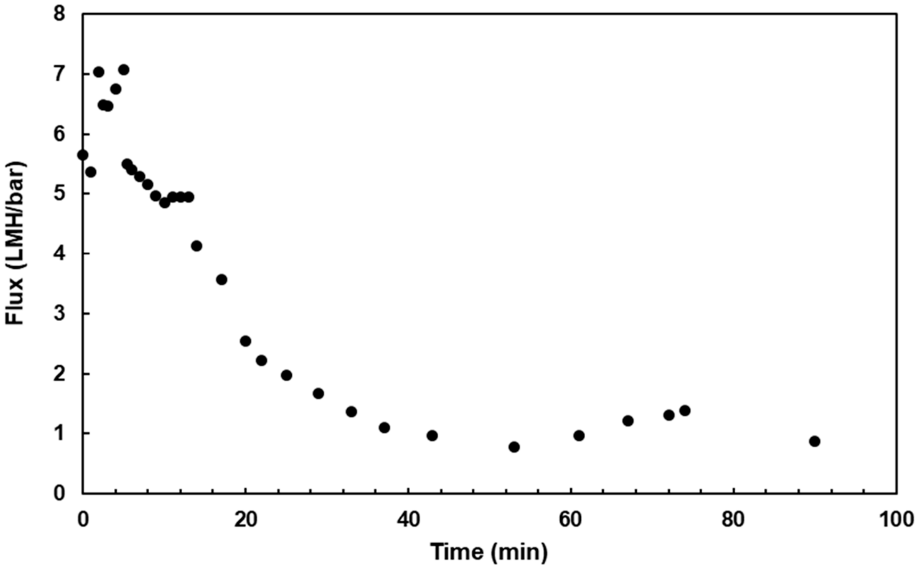

Figure S9: Compaction curve for E400 membrane in crossflow cell at 200PSI. No feed spacer is used.
